# Supplementary material for: A direct role for SNX9 in the biogenesis of filopodia
Source: J Cell Biol. 2020 Mar 25;219(4):e201909178. doi: 10.1083/jcb.201909178 (PMC7147113; doi:10.1083/jcb.201909178)
Supplement: Table S1 — shows statistics for effect of immunoblock and addition of SNX9 on FLS. [file JCB_201909178_TableS1.docx]

FLS count (> 3 µm)

|  | immunoblock vs control | |  | rescue vs control | |  | rescue vs immunoblock | |
| --- | --- | --- | --- | --- | --- | --- | --- | --- |
|  | p-value | significance |  | p-value | significance |  | p-value | significance |
| scFvFLS3 | 0.000217 | *** |  | 0.412717 | ns |  | 0.000217 | *** |
| scFvFLS4 | 0.000022 | *** |  | 0.595751 | ns |  | 0.000007 | *** |
| scFvFLS5 | 0.000003 | *** |  | 0.000655 | ** |  | 0.000018 | *** |
| scFvFLS21 | 0.000034 | *** |  | 0.000006 | *** |  | 0.000513 | ** |

Path length

|  | immunoblock vs control | |  | rescue vs control | |  | rescue vs immunoblock | |
| --- | --- | --- | --- | --- | --- | --- | --- | --- |
|  | p-value | significance |  | p-value | significance |  | p-value | significance |
| scFvFLS3 | 0.002057 | ** |  | 0.894332 | ns |  | 0.000217 | *** |
| scFvFLS4 | 0.000217 | *** |  | 0.633628 | ns |  | 0.000007 | *** |
| scFvFLS5 | 0.000034 | *** |  | 0.832588 | ns |  | 0.000034 | *** |
| scFvFLS21 | 0.000235 | *** |  | 0.000885 | ** |  | 0.000235 | *** |

**Sup Table 1**
