# Supplementary material for: A direct role for SNX9 in the biogenesis of filopodia
Source: J Cell Biol. 2020 Mar 25;219(4):e201909178. doi: 10.1083/jcb.201909178 (PMC7147113; doi:10.1083/jcb.201909178)
Supplement: Table S2 — shows statistics for effect of Wortmannin on FLS grown on different lipid compositions. [file JCB_201909178_TableS2.docx]

| Comparison | | |  | FLS length  (related to Fig. 3C) | |  | FLS count (> 3 µm)  (related to Fig. 3D) | |
| --- | --- | --- | --- | --- | --- | --- | --- | --- |
|  |  |  |  | p-value | significance |  | p-value | significance |
| PI(4,5)P_2_ only  DMSO | vs | PI(4,5)P_2_ only  wortmannin |  | <0.0001 | *** |  | <0.0001 | *** |
| PI(4,5)P_2_ only  DMSO | vs | +PI(3)P  wortmannin |  | <0.0001 | *** |  | <0.0001 | *** |
| PI(4,5)P_2_ only  DMSO | vs | +PI(3,4)P_2_  wortmannin |  | <0.0001 | *** |  | <0.0001 | *** |
| PI(4,5)P_2_ only  DMSO | vs | +PI(3,4,5)P_3_  wortmannin |  | <0.0001 | *** |  | <0.0001 | *** |
| PI(4,5)P_2_ only  wortmannin | vs | +PI(3)P  wortmannin |  | <0.0001 | *** |  | 0.0197 | * |
| PI(4,5)P_2_ only  wortmannin | vs | +PI(3,4)P_2_  wortmannin |  | 0.0247 | * |  | 0.5066 | ns |
| PI(4,5)P_2_ only  wortmannin | vs | +PI(3,4,5)P_3_  wortmannin |  | 0.0583 | ns |  | 0.7199 | ns |
| +PI(3)P  wortmannin | vs | +PI(3,4)P_2_  wortmannin |  | <0.0001 | *** |  | 0.0004 | *** |
| +PI(3)P  wortmannin | vs | +PI(3,4,5)P_3_  wortmannin |  | <0.0001 | *** |  | 0.0024 | ** |

**Sup Table 2**
